# Genome Sequencing of *Ralstonia solanacearum* CQPS-1, a Phylotype I Strain Collected from a Highland Area with Continuous Cropping of Tobacco

**DOI:** 10.3389/fmicb.2017.00974

**Published:** 2017-05-31

**Authors:** Ying Liu, Yuanman Tang, Xiyun Qin, Liang Yang, Gaofei Jiang, Shili Li, Wei Ding

**Affiliations:** ^1^Laboratory of Natural Products Pesticides, College of Plant Protection, Southwest UniversityChongqing, China; ^2^Yunnan Academy of Tobacco Agricultural ResearchYuxi, China; ^3^Laboratoire des Interactions Plantes-Microorganismes, Université de Toulouse, INRA, CNRSCastanet-Tolosan, France

**Keywords:** genome sequencing, *Ralstonia solanacearum*, virulence factors, type III effectors, comparative genomic analysis

## Abstract

*Ralstonia solanacearum*, an agent of bacterial wilt, is a highly variable species with a broad host range and wide geographic distribution. As a species complex, it has extensive genetic diversity and its living environment is polymorphic like the lowland and the highland area, so more genomes are needed for studying population evolution and environment adaptation. In this paper, we reported the genome sequencing of *R. solanacearum* strain CQPS-1 isolated from wilted tobacco in Pengshui, Chongqing, China, a highland area with severely acidified soil and continuous cropping of tobacco more than 20 years. The comparative genomic analysis among different *R. solanacearum* strains was also performed. The completed genome size of CQPS-1 was 5.89 Mb and contained the chromosome (3.83 Mb) and the megaplasmid (2.06 Mb). A total of 5229 coding sequences were predicted (the chromosome and megaplasmid encoded 3573 and 1656 genes, respectively). A comparative analysis with eight strains from four phylotypes showed that there was some variation among the species, e.g., a large set of specific genes in CQPS-1. Type III secretion system gene cluster (*hrp* gene cluster) was conserved in CQPS-1 compared with the reference strain GMI1000. In addition, most genes coding core type III effectors were also conserved with GMI1000, but significant gene variation was found in the gene *ripAA*: the identity compared with strain GMI1000 was 75% and the *hrp_II_* box promoter in the upstream had significantly mutated. This study provided a potential resource for further understanding of the relationship between variation of pathogenicity factors and adaptation to the host environment.

## Introduction

Plant bacterial wilt disease is caused by a soil-borne pathogen *Ralstonia solanacearum*, a complex species with extensive diversity (Hayward, 1991; Genin and Denny, 2012). It is widely distributed throughout the world and has a broad host range, including many dicotyledonous and monocotyledonous plants. Previously, *R. solanacearum* was subdivided into five races (based on the host range) and six biovars (based on their ability to metabolize disaccharides and hexose alcohols) (Buddenhagen et al., 1962; Hayward, 1964; Pegg and Moffett, 1971; He et al., 1983). Recently, it is divided into four phylotypes corresponding to its geographical origin: phylotype I from Asia, phylotype II from the Americas, phylotype III from Africa, and phylotype IV from the Indonesian archipelago (Fegan and Prior, 2005; Prior and Fegan, 2005). Moreover, the species complex has been divided into three species supported by genome analysis (Prior et al., 2016). Because of its highly diverse geographical distribution, host range, and genetic diversity, control of the pathogen is difficult, resulting in large economic losses (Hayward, 1991; Genin and Boucher, 2002; Genin and Denny, 2012).

To better understand the functions of pathogenicity determinants and the traits of aggressiveness under different ecological environments, the whole genome of *R. solanacearum* was sequenced. Phylotype I strain GMI1000 was the first strain subject to whole genome analysis (Salanoubat et al., 2002). Currently, there are 67 genomes in the National Center for Biotechnology Information (NCBI) database, of which more than ten genomes are complete (Salanoubat et al., 2002; Remenant et al., 2010, 2012; Li et al., 2011, 2016; Xu et al., 2011; Cao et al., 2013; Bocsanczy et al., 2014; Ailloud et al., 2015; She et al., 2015; Guarischi-Sousa et al., 2016). However, because of their high variation, more genome sequences are needed for analyzing the entire species.

Genomes are a very useful resource to understand the mechanism of plant–pathogen interaction and the phylogenetic analyses of the species. Ailloud et al. (2015) compared genomes of different strains to find an explanation for host range adaptation of *R. solanacearum* strains. In addition, the genome analysis also provided insight into the evolution of virulence, such as *hrp* gene clusters, and the type III effectors (T3Es) among *R. solanacearum* strains and other pathogenic bacteria (Genin and Boucher, 2004).

Phylotype I was one of the ongoing diversifying subspecies according to research focused on the evolutionary history of *R. solanacearum* using multilocus sequence analysis (MLSA) (Wicker et al., 2012). In China, phylotype I *R. solanacearum* strains infecting tobacco display sequevar diversity and are spreading from the lowlands to the highlands and cold areas (Liu et al., 2017). It is interesting to study the genetic variations of *R. solanacearum* influenced by highland circumstances and host environment. Here, we report the complete genome sequence of *R. solanacearum* CQPS-1, a strain isolate from a highland (>1000 m), where soil is severely acidified and tobacco has continuously cropped more than 20 years. Our goal is to explore the molecular traits that the bacterium uses to adapt to its environment and interact with plants. The genome comparison is performed to find dissimilarities between CQPS-1 and other phylotype I genomes as well as genomes of strains belonging to other phylotypes. Furthermore, in order to elucidate pathogenicity variations, the virulence factors of our sequence were compared with strain GMI1000. We found that type III secretion system (T3SS) gene cluster (*hrp* gene cluster) was conserved, and only some other T3Es had significant gene variations, which may be a result of the strain interacting with its host for a long time.

## Materials and Methods

### Strains and Genomic DNA Preparation

The *R. solanacearum* strain CQPS-1, belonging to phylotype I sequevar 17 (Liu et al., 2017), was isolated from a wilting tobacco plant (*Nicotiana tabacum*). The wilting plant was collected from Pengshui, Chongqing, China, where tobacco has been grown for more than 20 years; the elevation is more than 1000 m and the pH of soil is severely acidic (pH ≈ 5.0). Strains were grown at 30 ± 2°C in B liquid medium (Boucher et al., 1985). Genomic DNA was purified from overnight liquid cultures using the CTAB (hexadecyltrimethylammonium bromide) method (Wilson, 2001).

### Sequencing and Assembly

The whole genome was sequenced using the PacBio RS II platform with a 20-kb library. Reads were assembled using HGAP (version 2.3.0, Pacific Biosciences) (Chin et al., 2013). Assembly data for the complete genome have been deposited in GenBank with accession numbers CP016914 and CP016915 (chromosome and megaplasmid, respectively).

### Genome Components and Genome Annotation

CDS were predicted using Prodigal (Hyatt et al., 2010). A circular map of the genome was drawn by CIRCOS (Krzywinski et al., 2009). Genomic Islands (GIs) were predicted by using the GI prediction method IslandPath-DIOMB (Dhillon et al., 2015). Clustered regularly interspaced short palindromic repeat sequences (CRISPRs) were found using CRISPRFinder (Grissa et al., 2007b) and PILER-CR (Edgar, 2007). Functional annotation was based on BLASTp searches against the NCBI non-redundant (NR) database and the KEGG, Pfam, Swissprot, and TrEMBL databases. Cluster of Orthologous Group of proteins (COG) analysis was performed to generate functional annotations for coding sequences (reference to orthologous groups^1^) (Tatusov et al., 2001).

### The Virulence Dataset

Virulence factors were predicted based on the virulence factors database (VFDB^2^). The virulence factors of the *R. solanacearum* strain CQPS-1 analyzed in this study were selected according to Remenant et al. (2010). T3Es were annotated using the IANT “Ralstonia T3E” database (Peeters et al., 2013). Every gene annotation was then manually validated to ensure homogeneity of the start codon positions and to detect frameshifts and pseudogenization.

### Genomic Comparisons

The genome sequences of GMI1000, Y45, YC45, FQY_4, PO82, CFBP2957, CMR15, and PSI07 were downloaded from the NCBI and EMBL databases. Sequences were aligned using Clustal x (Jeanmougin et al., 1998). Phylogenetic analysis was performed using neighbor-joining (NJ) and the algorithm of Jukes and Cantor (1969) with 1,000 bootstrap resamplings in MEGA version 5 (Tamura et al., 2011). Nucleic acid co-linearity was performed using MCScanX according to the alignment results of homology relationships by BLAST (Altschul et al., 1997; Wang et al., 2012). The set of genes unique to strain CQPS-1 was found using OrthoMCL (Chen et al., 2006).

## Results

### Genome Features

**FIGURE 1:**
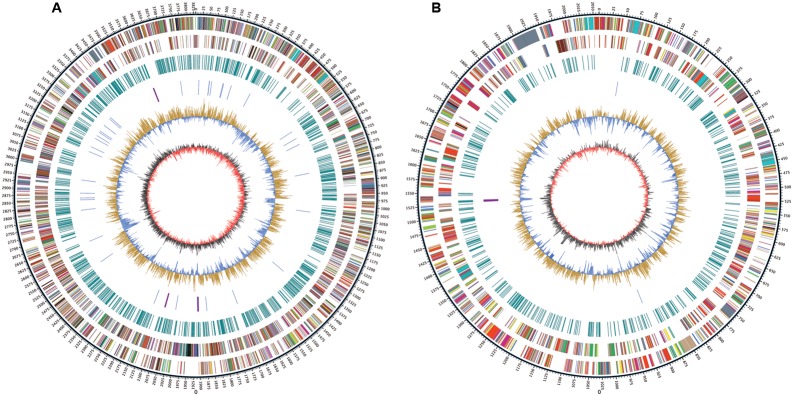
Circular map of *Ralstonia solanacearum* strain CQPS-1 genome. **(A)** Chromosome; **(B)** Megaplasmid. The distribution of the circle from outer to inner indicates genome size, forward CDS, reverse CDS, repeat sequences, tRNA (blue) and rRNA (purple), GC ratio (yellow means GC ratio of the region is higher than average GC ratio, blue means GC ratio of the region is lower than average GC ratio), and GC skew (gray represents a region with G content greater than C, red represents a region with C content greater than G).

Whole genome sequencing was performed with single molecule real-time sequencing (SMRT) on the PacBio RS II platform (Eid et al., 2009). The completed genome of *R. solanacearum* strain CQPS-1 was 5.89 Mb (GC%, 66.84%) and contained one circular chromosome (3.83 Mb, **Figure 1A**) and one megaplasmid (2.06 Mb, **Figure 1B**). The general features were shown in **Table 1**. The average GC content of the chromosome was 66.71% and that of the megaplasmid was 67.09%. A total of 5229 CDS were predicted (chromosome and megaplasmid encoded 3573 and 1656 genes, respectively). The CQPS-1 genome contained 12 rRNA and 58 tRNA.

**Table 1:** General features of the *Ralstonia solanacearum* strain CQPS-1 genome.

Attribute	Value
Genome size (Mp)	5.89
G+C ratio (%)	66.84
DNA coding (bp)	5,138,343
Protein coding genes	5229
rRNA	12
tRNA	58
Pseudo genes	23
Genes assigned to COGs	4700
Genomic Islands	21
CRISPR	9


### Genomic Islands and CRISPR Prediction

Genomic Islands are evidence of horizontal acquisition (Langille et al., 2010; Remenant et al., 2010). The GIs predicted in CQPS-1 are listed in Supplementary Table S1: a total of 21 GIs were predicted in the chromosome (13 GIs) and megaplasmid (8 GIs). CRISPRs can confer resistance to exogenous genetic elements such as phages and plasmids (Barrangou et al., 2007). To predict the CRISPRs of CQPS-1, the methods PILER-CR and CRISPRFinder were used. From the result predicted by the program PILER-CR, seven CRISPRs were found in the genome of CQPS-1; three were located in the chromosome, and four were in megaplasmid (Supplementary Table S2). Whereas two different questionable CRISPRs were predicted by using CRISPRFinder, one in the chromosome and another in the megaplasmid (Supplementary Table S2). Compared with the previous reports (Li et al., 2016), we knew that the putative CRISPR sequence in the chromosome of CQPS-1 (3,693,731-3,693,841) predicted by CRISPRFinder was completely conserved with the one located in the chromosome of strain GMI1000 (1,445,581-1,445,691).

### Genome Annotation

**FIGURE 2:**
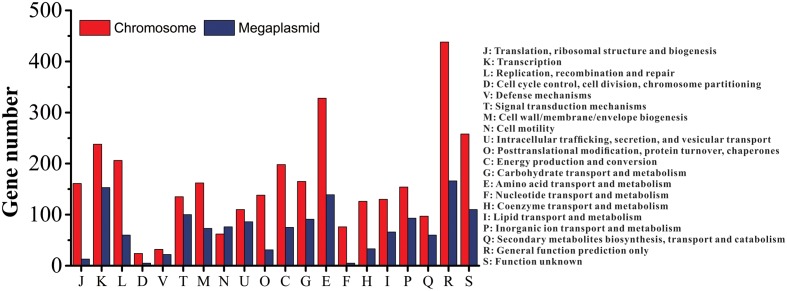
Distribution of genes with COG functional categories between the chromosome and the megaplasmid in strain CQPS-1.

Of the 5229 CDS, 4700 proteins can be assigned to 23 COG families (Supplementary Table S3). Except for the genes predicted to have general (604 genes) or unknown functions (368 genes), the largest group of genes were involved in amino acid transport and metabolism (467 genes, 8.93%). Compared to the distribution of genes in different COG families, the results showed that the megaplasmid had more genes than the chromosome in cell motility (**Figure 2**), which is consistent with a previous report by Li et al. (2016). In addition, a total of 2539 proteins had KEGG orthologs.

### Comparative Genome Analysis

**FIGURE 3:**
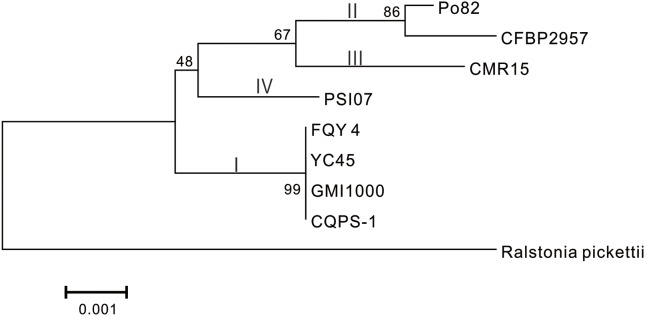
Phylogenetic tree of *R. solanacearum* strain CQPS-1 with other close species based on 16S rRNA. The tree was generated by MEGA-5 software using the neighbor-joining (NJ) and the algorithm of Jukes and Cantor (1969) with 1,000 bootstrap re-samplings. *Ralstonia pickettii* 12J (NCBI accession NC_010682) was used as an outgroup.

Phylogenetic tree was constructed using 16S rRNA. The result showed that CQPS-1 belonged to phylotype I, and was closest to strains YC45, FQY_4, and GMI1000 (**Figure 3**). When aligning syntenic genes of the CQPS-1 genome with other *R. solanacearum* genomes, the results demonstrated that the percentages of syntenic genes compared with phylotype I strains were more than other phylotype strains. The number of CDS in synteny with strain GMI1000 was highest (84.97%, **Table 2**), while the number of CDS in synteny with phylotype IIA strain CFBP2957 was the lowest (70.47%). According to the results of nucleic acid co-linearity, we know that there were a large number of inverse fragments among different *R. solanacearum* species, and many rearrangements were found in these genomes (**Table 2** and **Supplementary Figure S1**).

**Table 2:** The co-linearity results of strain CQPS-1compared to that of different *R. solanacearum* strains.

Compared strains	Percentage (%)^a^	Fragment numbers^b^	Average gene number^c^	Plus fragment^d^	Minus fragment^e^
GMI1000	84.97	42	105.79	16	26
Y45	84.11	46	95.61	24	22
YC45	80.13	121	34.63	61	60
FQY_4	83.74	44	99.52	23	21
PO82	71.31	59	63.20	30	29
CFBP2957	70.47	57	64.65	36	21
CMR15	75.92	38	104.47	28	10
PSI07	73.05	54	70.74	29	25


*^a^ The percentage of aligned gene number in all gene number of strain CQPS-1.*

*^b^The aligned fragments of strain CQPS-1 compared with other strains.*

*^c^The average gene numbers in one fragment.*

*^d^The number of plus aligned fragment.*

*^e^The number of minus aligned fragment.*

**FIGURE 4:**
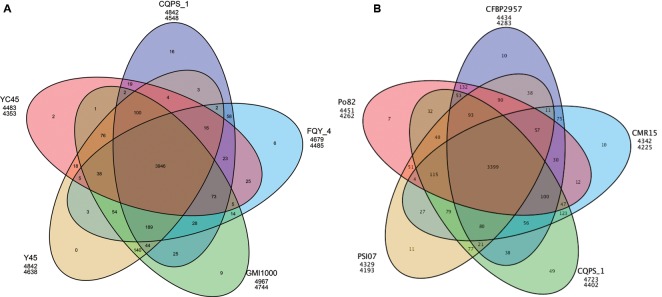
Venn diagram showing numbers of specific and shared gene families among five phylotype I species **(A)**, and five different phylotype species **(B)**. The number in the overlapping sections indicate shared numbers of gene families. The first line below each strain name represents the number of genes involved in the clustered gene families, and the second line represents the number of gene families that the strain has.

We also performed a pan-genomic analysis of *R. solanacearum* strains. First, we compared the genes of strain CQPS-1 to four phylotype I strains: GMI1000, YC45, Y45, and FQY_4. As shown in **Figure 4A**, 3946 gene families were involved in the core genome, which was shared by all compared strains. In addition, the number of specific gene families in strain CQPS-1 was 16 and contained 442 genes (specific gene numbers were shown in Supplementary Table S4). After annotation, the specific genes encoded a large number of hypothetical proteins and other proteins, such as transposase, LuxR family transcriptional regulator, signal peptide protein, membrane protein, T3E protein, etc. (detailed annotation data was shown in Supplementary Table S5). Then, strain CQPS-1, as a phylotype I strain, was compared with the other four phylotype strains (Po82, CFBP2957, CMR15, and PSI07). The results (shown in **Figure 4B**) showed that there were 3399 gene families shared by different phylotype strains. The number of CQPS-1-specific gene families was 49, including 625 genes (specific gene numbers were shown in Supplementary Table S6), most of which coded as hypothetical proteins (Supplementary Table S7).

### Virulence Factors

Potential virulence factors in the strain CQPS-1 were identified using the BLAST search in the VFDB database. A total of 622 putative virulence factors were aligned (the chromosome and megaplasmid had 363 and 259 genes, respectively). We also compared the virulence factors reported by Remenant et al. (2010) with strain GMI1000, including exopolysaccharide (EPS) biosynthetic genes, cell wall-degrading enzyme (CWDE) genes, response genes to the host defense and key virulence regulators. The results showed that these genes were highly identical with strain GMI1000 (Supplementary Table S8); the identities were more than 97%, except twitching motility gene *pilA*, whose identity was 91%.

### Comparison Analyses of Type III Secretion Systems and Type III Effectors

**FIGURE 5:**
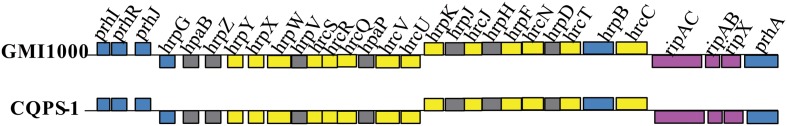
Genetic organization and gene comparison of *hrp* clusters between CQPS-1 and GMI1000.

Type III secretion systems, which has a syringe-like membrane structure and can inject T3Es into plant cells, causing disease or a hypersensitive response (HR), is important for the pathogenicity of *R. solanacearum* (Valls et al., 2006; Coll and Valls, 2013). T3SS is coded by hypersensitive response and pathogenicity (*hrp*) gene cluster, which is in the megaplasmid (Lindgren, 1997; Genin and Boucher, 2004). In strain CQPS-1, the *hrp* gene cluster contained 30 genes (spanning 29,682 bp, from 1,604,200 to 1,633,881). A comparison showed that the *hrp* gene cluster of CQPS-1 has a high similarity to that of the strain GMI1000 (the identity was 99%, **Figure 5**).

Type III effectors, presumed to modulate host innate immunity, are important virulence determinants for the pathogen (Poueymiro and Genin, 2009; Peeters et al., 2013). According to Peeters et al. (2013), 32 conserved or core T3Es have been defined. We compared the genes of 32 core T3Es of CQPS-1 to GMI1000. The results showed that 29 core T3Es genes were conserved (the coverage was 100%, and the identity was more than 90%), and some variations were found in others (**Table 3**). *RipB* had some base deletion, and the coverage was 93%. The identity of *ripG7* was 84%. *RipAA* (former name *avrA*), which can encode RipAA, the effector responsible for triggering HR on *N. tabacum* and *N. benthamiana* (Carney and Denny, 1990; Poueymiro et al., 2009), had 75% identity with strain GMI1000. In addition, there was a variation in the *hrp_II_* box promoter in the upstream of *ripAA*.

**Table 3:** Comparison of core type III effectors (T3Es) genes of strain CQPS-1 with strain GMI1000.

No.	Former effector name	New effector name	GMI1000 gene ID	CQPS-1 gene ID	Coverage (%)/Identity (%)
1	AWR2	RipA2	RSp0099	2_575	100/99
2	AWR4	RipA4	RSp0847	2_1346	100/99
3	AWR5	RipA5	RSp1024	2_1187	100/99
4	Rip2	RipB	Rsc0245	1_1159	93/99
5	Rip62	RipC1	Rsp1239	2_968	100/99
6	Rip34	RipD	RSp0304	2_770	100/95
7	Rip26	RipE1	Rsc3369	1_1488	100/99
8	PopF1	RipF1	Rsp1555	2_392	100/99
9	Gala5	RipG5	Rsc1801	1_3252	100/99
10	Gala7	RipG7	Rsc1357	1_3448	98/84
11	HLK1	RipH1	RSc1386	1_3419	100/99
12	HLK2	RipH2	RSp0215	2_686	100/97
13	HLK3	RipH3	RSp0160	2_628	100/99
14	Rip16	RipM	RSc1475	1_3332	100/99
15	PopS	RipR	Rsp1281	2_923	100/99
16	SKWP3	RipS3	RSp0930	2_1282	100/99
17	Rip59	RipU	RSp1212	2_993	100/99
18	PopW	RipW	Rsc2775	1_2087	100/99
19	PopA	RipX	Rsp0877	2_1316	100/98
20	Rip57	RipZ	RSp1031	2_1177	100/99
21	AvrA	RipAA	RSc0608	1_756	75/75
22	PopB	RipAB	Rsp0876	2_1317	100/99
23	PopC	RipAC	RSp0875	2_1318	100/99
24	Rip72	RipAD	RSp1601	2_433	100/99
25	Rip41	RipAI	RSp0838	2_1356	100/99
26	Rip21	RipAJ	RSc2101	1_2771	100/99
27	Brg40	RipAM	RSc3272	1_1588	100/99
28	Rip43	RipAN	Rsp0845	2_1348	100/99
29	Rip50	RipAO	RSp0879	2_1314	100/99
30	Rip51	RipAQ	RSp0885	2_1307	100/99
31	Rip61	RipAR	RSp1236	2_971	100/99
32	Rip55	RipAY	RSp1022	2_1190	100/98


## Discussion

*Ralstonia solanacearum*, which causes very large economic losses every year in China, is spreading to high altitudes and cold areas (Liu et al., 2017). This study presented a complete genome of the *R. solanacearum* strain CQPS-1 collected from a highland area with severely acidified soil and continuous cropping of tobacco. The technology used for sequencing the genome was SMRT (Eid et al., 2009; McCarthy, 2010), which is applied to finished microbial genomes by Pacific Biosciences due to its longer read length. The genome contained a 3.83 Mb chromosome and a 2.06 Mb megaplasmid. A comparative genomics analysis was also performed to identify the differences between strain CQPS-1 and other representative strains. From the results, we found that the genome of strain CQPS-1 showed some degree of variation, which could provide some evidence for a relationship between effectors variance and pathogen adaptation to host and environment.

Phylogenetic analysis was performed based on 16S rRNA. From the result, we knew that strain CQPS-1 was more similar to other phylotype I strains, such as GMI1000, YC45, and FQY_4, than other phylotypes, such as CFBP2957 (phylotype IIA), Po82 (phylotype IIB), CMR15 (phylotype III), and PSI07 (phylotype IV). Co-linearity also supported the result. Genome synteny, which studies the conserved multigene regions, is useful to assess species evolution and predict the gene function (Suyama and Bork, 2001; Bentley and Parkhill, 2004). According to our result of co-linearity analysis, there were different levels of inverse fragments and dissimilarities among these phylotype I strains and different phylotype strains. Remenant et al. (2010) demonstrated that the *R. solanacearum* genomes were highly syntenic when working on six strains, in addition, intra- and inter-replicon rearrangements occurred in the history of the organisms. In bacteria, rapid evolutionary changes such as chromosomal rearrangements always accompanied by host restriction (Moran and Plague, 2004).

Another important mechanisms in the evolution of pathogens is horizontal gene transfer (HGT) (Bhattacharya et al., 2003; Guidot et al., 2009). Bacteria could get genes from other different species such as archaea, bacteriophage, and eukaryotes (Koonin et al., 2001). *R. solanacearum* can transfer genes to adapt to novel ecological niches (Guidot et al., 2009). GIs, which known as pathogenicity islands, were thought to be the result of HGT (Langille et al., 2010). There were 21 GIs found in the genome of CQPS-1. The details of these GIs need to be further analyzed. Our complete genome can supply the resource to explore the species evolution interacted with different host plants and study the HGT of *R. solanacearum* strains occurring in nature.

There were nine CRISPRs predicted in CQPS-1 genome by using two methods, PILER-CR and CRISPRFinder. PILER-CR is a fast and accurate program based on an elegant algorithm to identify the CRISPR properties (Edgar, 2007), and CRISPRFinder is chosen because it can find very small CRISPRs (contained less than three, three or seven spacers) (Grissa et al., 2007a). The results predicted by the two methods showed that there were no intersection, and two small CRISPRs were found by CRISPRFinder.

Pan-genomic analysis of phylotype I strains demonstrated that the numbers of specific genes among compared phylotype I strains were different (ranging from 30 to 478). Phylotype I, of East African/Asian origin, can infect the largest number of host plants (Hayward, 1994). Strain GMI1000 has been isolated from tomatoes (Boucher et al., 1985), strains Y45 and FQY_4 have been known to infect tobacco (Li et al., 2011; Cao et al., 2013), and strain YC45 has been collected from ginger plants (She et al., 2015). The variety of host environments may be one of the reasons that this lineage is highly divergent. The function of specific genes should be further analyzed in depth to understand the relationship between specific genes and the adaptation of strains. For example, several genes encoded T3E proteins were found among specific genes in CQPS-1 when compared with other phylotype I strains (Supplementary Table S5). Whether these genes work is still unknown.

According to our results, the *hrp* gene cluster of CQPS-1 was conserved compared with GMI1000, which is consistent with the previous report that *hrp* cluster was highly conserved among phylotype I strains (Li et al., 2016). T3Es, translocated by T3SS, are highly variable and may play a role in shaping or extending the host range of strains according to previous studies (Castaneda et al., 2005; Hajri et al., 2009; Genin, 2010; Baltrus et al., 2011). Furthermore, they could co-evolve with the plant targets, such as the effector RipG7, the essential determinant of *R. solanacearum* strains for virulence on the legume plant *Medicago truncatula* (Wang et al., 2016). In this study, we found that the *ripAA* of strain CQPS-1 was variable compared with that of GMI1000: only 75% was identical with the gene of strain GMI1000, and there was a significant variation in the *hrp_II_* box promoter of *ripAA* in strain CQPS-1. It is known that the RipAA (AvrA) of GMI1000 is the major determinant causing HR on *N. tabacum* and *N. benthamiana* (Poueymiro et al., 2009). Strain CQPS-1 was collected from a location where tobacco has been grown for more than 20 years. We speculated that the mutative *ripAA* may be one of the results of an effector interacting with tobacco for a long time, which could help pathogen to avoid host recognition. This result provided another parameter to analyze effectors co-evolving with hosts.

In summary, this study showed the whole genome of strain CQPS-1 and comparative genomics analyses among different *R. solanacearum* strains. The genome variability presumably plays an important role when *R. solanacearum* strains adapt themselves to a host environment, which could provide an essential platform for studying plant–pathogen interactions for a long time.

## Author Contributions

Experimental design and authorship: YL and WD; Experiments and data analysis: YL, YT, LY, GJ, and SL; Manuscript revised: GJ, WD, and XQ; All authors read and approved the final manuscript.

## Conflict of Interest Statement

The authors declare that the research was conducted in the absence of any commercial or financial relationships that could be construed as a potential conflict of interest.

## Footnotes

1 http://www.ncbi.nlm.nih.gov/COG

2 http://www.mgc.ac.cn/VFs/

## Supplementary Material

**FIGURE S1 |** Nucleic acid co-linearity of strain CQPS-1 vs. their orthologs, **(A)** GMI1000, **(B)** Y45, **(C)** YC45, **(D)** FQY_4, **(E)** PO82, **(F)** CFBP2957, **(G)** CMR15, and **(H)** PSI07, respectively.
