# Supplementary material for: Genome Sequencing of Ralstonia solanacearum CQPS-1, a Phylotype I Strain Collected from a Highland Area with Continuous Cropping of Tobacco
Source: Front Microbiol. 2017 May 31;8:974. doi: 10.3389/fmicb.2017.00974 (PMC5449461; doi:10.3389/fmicb.2017.00974)
Supplement: Supplementary file 2 [file Table_1.DOCX]

Supplementary Material

**Genome Sequencing of *Ralstonia solanacearum* CQPS-1, a Phylotype I Strain Collected from highland area with severely acidified soil**

**Ying Liu, Yuanman Tang, Liang Yang, Gaofei Jiang, Shili Li, Wei Ding***

* **Correspondence:** Corresponding Author: dingw@swu.edu.cn

**Supplementary Table S1** Genomic Islands of *Ralstonia solanacearum* strains CQPS-1.

| No. | Location | Start | End | Length |
| --- | --- | --- | --- | --- |
| 1 | Chromosome | 428525 | 459566 | 31042 |
| 2 | Chromosome | 986787 | 1012338 | 25552 |
| 3 | Chromosome | 1180790 | 1193487 | 12698 |
| 4 | Chromosome | 1258067 | 1278681 | 20615 |
| 5 | Chromosome | 1689453 | 1699588 | 10136 |
| 6 | Chromosome | 1859197 | 1868432 | 9236 |
| 7 | Chromosome | 2272498 | 2287934 | 15437 |
| 8 | Chromosome | 2441192 | 2448117 | 6926 |
| 9 | Chromosome | 2456048 | 2461823 | 5776 |
| 10 | Chromosome | 2526333 | 2538614 | 12282 |
| 11 | Chromosome | 2594919 | 2632704 | 37786 |
| 12 | Chromosome | 3103246 | 3109052 | 5807 |
| 13 | Chromosome | 3141932 | 3150729 | 8798 |
| 14 | Megaplasmid | 547988 | 551133 | 3146 |
| 15 | Megaplasmid | 1059495 | 1081996 | 22502 |
| 16 | Megaplasmid | 1276812 | 1287027 | 10216 |
| 17 | Megaplasmid | 1757313 | 1766102 | 8790 |
| 18 | Megaplasmid | 1826645 | 1834201 | 7557 |
| 19 | Megaplasmid | 2012868 | 2025113 | 12246 |
| 20 | Megaplasmid | 2027661 | 2031890 | 4230 |
| 21 | Megaplasmid | 2034871 | 2051999 | 17129 |
