# Supplementary material for: Genome Sequencing of Ralstonia solanacearum CQPS-1, a Phylotype I Strain Collected from a Highland Area with Continuous Cropping of Tobacco
Source: Front Microbiol. 2017 May 31;8:974. doi: 10.3389/fmicb.2017.00974 (PMC5449461; doi:10.3389/fmicb.2017.00974)
Supplement: Supplementary file 3 [file Table_2.DOCX]

Supplementary Material

**Genome Sequencing of *Ralstonia solanacearum* CQPS-1, a Phylotype I Strain Collected from a Highland Area with Severely Acidified Soil**

**Ying Liu, Yuanman Tang, Liang Yang, Gaofei Jiang, Shili Li, Wei Ding***

* **Correspondence:** Corresponding Author: dingw@swu.edu.cn

**Supplementary Table S2** Information about CRISPRs in genome of strains CQPS-1.

| Method | PILER-CR | | CRISPRFinder | |
| --- | --- | --- | --- | --- |
| Location | Chromosome | Megaplasmid | Chromosome | Megaplasmid |
| Start- End | 111,590-111,765 | 125,054-125,123 | 3,693,731-3,693,841 | 1,058,474-1,058,631 |
|  | 847,211-847,355 | 1,242,234-1,242,360 |  |  |
|  | 2,175,582-2,175,912 | 1,716,685-1,716,766 |  |  |
|  |  | 1,783,067-1,783,137 |  |  |
