# Supplementary material for: Genome Sequencing of Ralstonia solanacearum CQPS-1, a Phylotype I Strain Collected from a Highland Area with Continuous Cropping of Tobacco
Source: Front Microbiol. 2017 May 31;8:974. doi: 10.3389/fmicb.2017.00974 (PMC5449461; doi:10.3389/fmicb.2017.00974)
Supplement: Supplementary file 4 [file Table_3.DOCX]

Supplementary Material

**Genome Sequencing of *Ralstonia solanacearum* CQPS-1, a Phylotype I Strain Collected from a Highland Area with Severely Acidified Soil**

**Ying Liu, Yuanman Tang, Liang Yang, Gaofei Jiang, Shili Li, Wei Ding***

* **Correspondence:** Corresponding Author: dingw@swu.edu.cn

**Supplementary Table S3** The number of genes with COG functional categories between the chromosome and the megaplasmid in strain CQPS-1.

| COG Class_Description | Number of chromosome | Number of megaplasmid | Total | Percentage(%) |
| --- | --- | --- | --- | --- |
| Translation, ribosomal structure and biogenesis | 161 | 13 | 174 | 3.33 |
| RNA processing and modification | 2 | 0 | 2 | 0.04 |
| Transcription | 238 | 153 | 391 | 7.48 |
| Replication, recombination and repair | 206 | 60 | 266 | 5.09 |
| Chromatin structure and dynamics | 2 | 0 | 2 | 0.04 |
| Cell cycle control, cell division, chromosome partitioning | 24 | 5 | 29 | 0.55 |
| Defense mechanisms | 32 | 22 | 54 | 1.03 |
| Signal transduction mechanisms | 135 | 100 | 235 | 4.49 |
| Cell wall/membrane/envelope biogenesis | 162 | 73 | 235 | 4.49 |
| Cell motility | 62 | 76 | 138 | 2.64 |
| Extracellular structures | 0 | 1 | 1 | 0.02 |
| Intracellular trafficking, secretion, and vesicular transport | 110 | 86 | 196 | 3.75 |
| Posttranslational modification, protein turnover, chaperones | 138 | 31 | 169 | 3.23 |
| Energy production and conversion | 198 | 75 | 273 | 5.22 |
| Carbohydrate transport and metabolism | 165 | 91 | 256 | 4.90 |
| Amino acid transport and metabolism | 328 | 139 | 467 | 8.93 |
| Nucleotide transport and metabolism | 76 | 5 | 81 | 1.55 |
| Coenzyme transport and metabolism | 126 | 33 | 159 | 3.04 |
| Lipid transport and metabolism | 130 | 66 | 196 | 3.75 |
| Inorganic ion transport and metabolism | 154 | 93 | 247 | 4.72 |
| Secondary metabolites biosynthesis, transport and catabolism | 97 | 60 | 157 | 3.00 |
| General function prediction only | 438 | 166 | 604 | 11.55 |
| Function unknown | 258 | 110 | 368 | 7.04 |
