# Supplementary material for: Genome Sequencing of Ralstonia solanacearum CQPS-1, a Phylotype I Strain Collected from a Highland Area with Continuous Cropping of Tobacco
Source: Front Microbiol. 2017 May 31;8:974. doi: 10.3389/fmicb.2017.00974 (PMC5449461; doi:10.3389/fmicb.2017.00974)
Supplement: Supplementary file 5 [file Table_4.DOCX]

Supplementary Material

**Genome Sequencing of *Ralstonia solanacearum* CQPS-1, a Phylotype I Strain Collected from a Highland Area with Severely Acidified Soil**

**Ying Liu, Yuanman Tang, Liang Yang, Gaofei Jiang, Shili Li, Wei Ding***

* **Correspondence:** Corresponding Author: dingw@swu.edu.cn

**Supplementary Table S4** The number of shared genes and specific genes in 5 phylotype I strains

| Strain | Share gene number | Specific gene number |
| --- | --- | --- |
| CQPS_1 | 4,787 | 442 |
| FQY_4 | 4,661 | 478 |
| GMI1000 | 4,949 | 171 |
| Y45 | 4,842 | 30 |
| YC45 | 4,472 | 149 |
