# Supplementary material for: Genome Sequencing of Ralstonia solanacearum CQPS-1, a Phylotype I Strain Collected from a Highland Area with Continuous Cropping of Tobacco
Source: Front Microbiol. 2017 May 31;8:974. doi: 10.3389/fmicb.2017.00974 (PMC5449461; doi:10.3389/fmicb.2017.00974)
Supplement: Supplementary file 7 [file Table_6.DOCX]

Supplementary Material

**Genome Sequencing of *Ralstonia solanacearum* CQPS-1, a Phylotype I Strain Collected from a Highland Area with Severely Acidified Soil**

**Ying Liu, Yuanman Tang, Liang Yang, Gaofei Jiang, Shili Li, Wei Ding***

* **Correspondence:** Corresponding Author: dingw@swu.edu.cn

**Supplementary Table S6** The number of shared genes and specific genes in different phylotype strains

| Strain | Share gene number | Specific gene number |
| --- | --- | --- |
| CFBP2957 | 4,402 | 617 |
| CMR15 | 4,311 | 668 |
| CQPS_1 | 4,604 | 625 |
| PSI07 | 4,304 | 674 |
| Po82 | 4,433 | 585 |
