# Supplementary material for: Genome Sequencing of Ralstonia solanacearum CQPS-1, a Phylotype I Strain Collected from a Highland Area with Continuous Cropping of Tobacco
Source: Front Microbiol. 2017 May 31;8:974. doi: 10.3389/fmicb.2017.00974 (PMC5449461; doi:10.3389/fmicb.2017.00974)
Supplement: Supplementary file 9 [file Table_8.DOCX]

Supplementary Material

**Genome Sequencing of *Ralstonia solanacearum* CQPS-1, a Phylotype I Strain Collected from a Highland Area with Severely Acidified Soil**

**Ying Liu, Yuanman Tang, Liang Yang, Gaofei Jiang, Shili Li, Wei Ding***

* **Correspondence:** Corresponding Author: dingw@swu.edu.cn

**Supplementary Table S8** Identity (%) comparison with virulence genes identified in *Ralstonia solanacearum* CQPS-1 genome and GMI1000

| No. | Classify | Gene name | Gene ID | | Identity |
| --- | --- | --- | --- | --- | --- |
|  |  |  | CQPS-1 | GMI1000 |  |
| 1 | EPS biosynthetic genes | *epsA* | 2_1193 | RSp1020 | 99.00% |
| 2 |  | *epsP* | 2_1194 | RSp1019 | 99.00% |
| 3 |  | *epsB* | 2_1195 | RSp1018 | 99.00% |
| 4 |  | *epsC* | 2_1196 | RSp1017 | 99.00% |
| 5 |  | *epsD* | 2_1197 | RSp1016 | 99.00% |
| 6 |  | *epsE* | 2_1198 | RSp1015 | 99.00% |
| 7 |  | *epsF* | 2_1199 | RSp1014 | 99.00% |
| 8 |  | *epsR* | 2_806 | RSp0338 | 99.00% |
| 9 | Cell Wall Degrading Enzymes (CWDE) | *pehB* | 1_3208 | RSc1756 | 99.00% |
| 10 |  | *pehC* | 2_1361 | RSp0833 | 99.00% |
| 11 |  | *egl* | 2_630 | RSp0162 | 99.00% |
| 12 |  | *plcN* | 1_1084 | RSc0319 | 99.00% |
| 13 | Response to host defenses | *katE* | 2_418 | RSp1581 | 99.00% |
| 14 |  | *oxyR* | 1_2184 | RSc2690 | 99.00% |
| 15 |  | *dinF* | 2_753 | RSp0283 | 99.00% |
| 16 |  | *acrA* | 1_1414 | RSc0011 | 98.00% |
| 17 |  | *acrB* | 1_1416 | RSc0010 | 99.00% |
| 18 | Key virulence regulators | *phcA* | 1_2113 | RSc2748 | 99.00% |
| 19 |  | *phcB* | 1_2127 | RSc2735 | 99.00% |
| 20 |  | *vsrA* | 1_1113 | RSc0289 | 99.00% |
| 21 |  | *vsrD* | 1_1110 | RSc0292 | 99.00% |
| 22 |  | *vsrB* | 1_2351 | RSc2456 | 99.00% |
| 23 |  | *vsrC* | 1_2352 | RSc2455 | 99.00% |
| 24 |  | *xpsR* | 2_1211 | RSp1003 | 99.00% |
| 25 |  | *pehS* | 1_2056 | RSc2808 | 99.00% |
| 26 |  | *pehR* | 1_2057 | RSc2807 | 99.00% |
| 27 |  | *solI* | 1_1573 | RSc3286 | 99.00% |
| 28 |  | *solR* | 1_1572 | RSc3287 | 99.00% |
| 29 | Chemotaxis | *cheA* | 2_246 | RSp1408 | 99.00% |
| 30 |  | *cheW* | 2_245 | RSp1407 | 99.00% |
| 31 | Swimming motility | *fliC* | 2_851 | RSp0382 | 99.00% |
| 32 |  | *flgM* | 2_808 | RSp0340 | 100.00% |
| 33 | Twitching motility | *pilA* | 1_806 | RSc0558 | 91.00% |
| 34 |  | *pilP* | 1_1886 | RSc2972 | 100.00% |
